# Multiple DNA repair pathways prevent acetaldehyde-induced mutagenesis in yeast

**DOI:** 10.1093/genetics/iyae213

**Published:** 2024-12-21

**Authors:** Latarsha Porcher, Sriram Vijayraghavan, Yashvi Patel, Samuel Becker, Thomas Blouin, James McCollum, Piotr A Mieczkowski, Natalie Saini

**Affiliations:** Department of Biochemistry and Molecular Biology, Medical University of South Carolina, Charleston, SC 29425, USA; Department of Biochemistry and Molecular Biology, Medical University of South Carolina, Charleston, SC 29425, USA; Department of Biochemistry and Molecular Biology, Medical University of South Carolina, Charleston, SC 29425, USA; Department of Biochemistry and Molecular Biology, Medical University of South Carolina, Charleston, SC 29425, USA; Department of Biochemistry and Molecular Biology, Medical University of South Carolina, Charleston, SC 29425, USA; Department of Biochemistry and Molecular Biology, Medical University of South Carolina, Charleston, SC 29425, USA; Department of Genetics, Lineberger Comprehensive Cancer Center, University of North Carolina at Chapel Hill, Chapel Hill, NC 27599, USA; Department of Biochemistry and Molecular Biology, Medical University of South Carolina, Charleston, SC 29425, USA

**Keywords:** acetaldehyde, nucleotide excision repair, DNA protein crosslink repair, mutagenesis

## Abstract

Acetaldehyde is the primary metabolite of alcohol and is present in many environmental sources, including tobacco smoke. Acetaldehyde is genotoxic, whereby it can form DNA adducts and lead to mutagenesis. Individuals with defects in acetaldehyde clearance pathways have increased susceptibility to alcohol-associated cancers. Moreover, a mutation signature specific to acetaldehyde exposure is widespread in alcohol- and smoking-associated cancers. However, the pathways that repair acetaldehyde-induced DNA damage and thus prevent mutagenesis are vaguely understood. Here, we used *Saccharomyces cerevisiae* to delete genes in each of the major DNA repair pathways to identify those that alter acetaldehyde-induced mutagenesis. We observed that loss of functional nucleotide excision repair had the largest effect on acetaldehyde mutagenesis. In addition, base excision repair and DNA protein crosslink repair pathways were involved in modulating acetaldehyde mutagenesis, while mismatch repair, homologous recombination, and postreplication repair are dispensable for acetaldehyde mutagenesis. Acetaldehyde-induced mutations in a nucleotide excision repair–deficient (*Δrad1*) background were dependent on translesion synthesis and DNA interstrand crosslink repair. Moreover, whole-genome sequencing of the mutated isolates demonstrated an increase in C→A changes coupled with an enrichment of gCn→A changes, which is diagnostic of acetaldehyde exposure in yeast and in human cancers. Finally, downregulation of the leading strand replicative polymerase Pol epsilon, but not the lagging strand polymerase, resulted in increased acetaldehyde mutagenesis, indicating that lesions are likely formed on the leading strand. Our findings demonstrate that multiple DNA repair pathways coordinate to prevent acetaldehyde-induced mutagenesis.

## Introduction

Acetaldehyde is produced as a metabolic byproduct in response to numerous exogenous agents, including tobacco smoke, air pollution, and food products, and is a major product of alcohol metabolism (Lin and Greenberg 1954; Cheng 2010). Individuals who are unable to metabolize acetaldehyde, such as those carrying mutations in the aldehyde dehydrogenase 2 gene (*ALDH2*) have an elevated risk for alcohol- or smoking-related carcinogenesis (Yang *et al.* 2007; Brooks *et al.* 2009; Cui *et al.* 2009; Wei *et al.* 2021). Further, mice with defects in *ALHD2* and the Fanconi anemia pathway also demonstrate increased predisposition to leukemia (Langevin *et al.* 2011). Based on these data, the US Environmental Protection Agency currently classifies acetaldehyde as a group B2 carcinogen (Agency 1987, 1999).

Genome instability is the primary mechanism by which acetaldehyde exposure has been hypothesized to promote carcinogenesis. Biochemically, acetaldehyde can directly form a variety of DNA lesions, including *N*^2^-ethylidene-2′-deoxyguanosine (*N*^2^-Eti-dG), 1,*N*^2^-propano-2′-deoxyguanosine, and G-G inter- and intrastrand crosslinks (Matsuda *et al.* 1998; Brooks and Theruvathu 2005; Brooks and Zakhari 2014; Balbo and Brooks 2015; Sonohara *et al.* 2019). Such adducts have been detected in individuals upon alcohol consumption or acetaldehyde exposure (Chen *et al.* 2007; Balbo *et al.* 2012). How these lesions are repaired and whether they contribute to mutagenesis are poorly understood.

Interestingly, the prevalence and mechanism of acetaldehyde-associated mutagenicity are highly debated. Studies in yeast (Voordeckers *et al.* 2020) and in human-induced pluripotent stem cells (iPSCs) (Kucab *et al.* 2019) determined that acetaldehyde is not mutagenic. Contrary to these studies, we recently demonstrated that acetaldehyde is a single-stranded DNA mutagen in yeast (Vijayraghavan *et al.* 2022). Acetaldehyde exposure led to an increase in C→A mutations in a gCn context (mutated residue is capitalized, *n* = a, t, g, or c) in yeast strains. The observed mutagenesis was found to be dependent on the presence of single-stranded DNA in yeast wherein DNA repair is not functional (Vijayraghavan *et al.* 2022). This gCn→A signature was further identified in tumors associated with smoking or alcohol consumption in whole-genome–sequenced cohorts from the Pan-cancer Analysis of Whole Genomes (ICGC/TCGA Pan-Cancer Analysis of Whole Genomes Consortium 2020) and whole-exome–sequenced cohorts from the International Cancer Genome Consortium (International Cancer Genome Consortium *et al.* 2010; Vijayraghavan *et al.* 2022). While our findings indicate widespread acetaldehyde-induced mutagenesis, it is likely that substrate limitations and highly efficient DNA repair constrain the detection of other relevant acetaldehyde-associated mutations.

Several studies have highlighted the DNA repair pathways that render cells more susceptible to the effects of acetaldehyde exposure. Mice deficient in ALDH2 and FANCD2 had a higher mutation load in hematopoietic stem cells due to the accumulation of endogenous DNA damage (Garaycoechea *et al.* 2018). FANCD2 was also implicated in resolving interstrand crosslinks (ICLs) induced by acetaldehyde in oral keratinocytes (Peake *et al.* 2021). The Fanconi anemia pathway was also found to be responsible for repair of DNA intrastrand crosslinks created in vitro on plasmids (Hodskinson *et al.* 2020). These studies show that the Fanconi anemia pathway plays a key role in preventing acetaldehyde-induced genome instability.

Similarly, mismatch repair (MMR) has also previously been shown to increase acetaldehyde-induced genome instability. *ALDH1B1* and *MSH2* deletion in mice also led to increased colonic tumors, likely due to elevated endogenous acetaldehyde levels (Cerretelli *et al.* 2023). Similarly, cell extracts deficient in xeroderma pigmentosum group A (XPA) proteins or human cell lines with XPA defects were unable to repair acetaldehyde-induced DNA damage on plasmid DNA, indicating a role of nucleotide excision repair (NER) in repair of acetaldehyde DNA damage (Sonohara *et al.* 2022). Finally, in *Schizosaccharomyces pombe*, defects in homologous recombination (HR), aldehyde clearance pathways, NER, fork protection complex, and checkpoint response pathways were found to sensitize cells to exogenous acetaldehyde (Noguchi *et al.* 2017). However, because these studies primarily focused on DNA damage and cell death as the primary outcomes of acetaldehyde exposure as opposed to mutagenesis, by and large, the genetic determinants of acetaldehyde-induced mutagenesis are poorly understood.

Here, we tested the roles of various DNA repair and replication pathways in yeast to identify those that function to prevent mutagenesis upon acetaldehyde exposure. Similar to previous studies, we demonstrate that acetaldehyde exposure is not mutagenic in DNA repair proficient yeast. However, deficiencies in NER, base excision repair (BER), translesion synthesis (TLS), ICL repair, DNA protein crosslink (DPC) repair, and DNA replication, either alone or in combination, were found to modulate acetaldehyde mutagenesis. As such, our work demonstrates that multiple DNA repair pathways can efficiently remove DNA damage induced by acetaldehyde, thus preventing mutagenesis.

## Materials and methods

### Strains

Yeast strains used in this study are derived from the ySR128 (*MATα ura3Δ can1Δ ade2Δ leu2-3,112 trp1-289 ChrV:lys2:ADE2-URA3-CAN1*). Deletions of the genes *RAD1, RAD14, APN2, WSS1, DDI1, PSO2, REV3, and OGG1* were performed using either *KANMX* or *HPHMX* cassettes, conferring resistance to G418 and hygromycin, respectively. *APN1* was deleted using the *bsd* gene conferring resistance to blasticidin. The complete strain list is provided in Supplementary Table 5. The tetracycline promoter construct was obtained from Euroscarf (pCM225). PCR was performed with primers carrying overhangs for *POL2* and *POL3* promoter regions, and 1-step integration was used to replace the promoters for *POL* and *POL3.* The promoter is tagged with the *KANMX* gene.

### Acetaldehyde treatment and mutation frequency measurement

Yeast strains were incubated at 30°C shaking at 160 rpm overnight. The next day, the yeast cells were counted on a hemocytometer and diluted 1:10 in 30 mL of fresh media to yield a starting concentration of 1 × 10^7^ cells/mL. The 30-mL yeast cultures were incubated at 30°C with shaking at 160 rpm for 3 h. The cultures were then spun in 50-mL conical tubes at 2,500 rpm for 5 min, washed once with water, and then split into two 15-mL samples in 15-mL conical tubes. One set of the 15-mL conical tubes was treated as controls (i.e. water only), whereas the second set was treated with 1% acetaldehyde (Sigma, catalog number 402788) diluted in water. Acetaldehyde was kept chilled, and chilled pipet tips were used to prevent evaporation. Both sets of tubes were incubated for 1 h at 30°C in a rotary shaker. The cultures were then spun down and washed once with water to remove acetaldehyde and resuspended in 2 mL of water. Appropriate dilutions of cells were plated on synthetic complete (SC) media (MP Biomedicals) to measure viability and SC-arginine plates containing 60 mg/mL of canavanine (Sigma). Plates were incubated at 30°C for 5 days. Colonies were counted using the aCOLyte 3 automated colony counter (Synbiosis).

*CAN1* in our yeast strains is a counter-selectable marker, and mutations that inactivate the gene render yeast strains resistant to canavanine. The mutation frequency was calculated using the formula:


$$\frac{\mathrm{Canavanine}-\mathrm{resistant}\;\mathrm{colonies}\;\mathrm{per}\;\mathrm{mL}}{\mathrm{Viable}\;\mathrm{colonies}\;\mathrm{per}\;\mathrm{mL}}$$

A Mann–Whitney U test was performed, and mutation frequencies were compared between untreated and treated cultures for the same genotypes. *q*-values were calculated using the Benjamini–Hochberg method across all mutation frequency assays done in this study. The resulting *P*- and *q*-values are presented in Supplementary Table 1c.

Cellular viability after acetaldehyde treatment was calculated as:


$$\frac{100\times \mathrm{viable}\;\mathrm{colonies}\;\mathrm{per}\;\mathrm{mL}\;\mathrm{after}\;\mathrm{acetaldehyde}\;\mathrm{treatment}}{\mathrm{Viable}\;\mathrm{colonies}\;\mathrm{per}\;\mathrm{mL}\;\mathrm{in}\;\mathrm{water}-\mathrm{treated}\;\mathrm{samples}}$$

We annotate water-treated isolates at 100% viability. All acetaldehyde-treated isolates are compared with their respective water-treated cultures.

Mann–Whitney U test was performed, and viability of cultures was compared against the wild-type strains. *q-*values were calculated using the Benjamini–Hochberg method across all viability assays done in this study. The resulting *P*- and *q*-values are presented in Supplementary Table 2b.

### Quantification of gene expression for tetracycline-downregulatable constructs

Total RNA was extracted from the *tet-POL2* and *tet-POL3* yeast cultures as well as wild-type yeast cultures wherein the polymerases were under their native promoters using the YeaStar RNA Kit (Zymo Research). Cultures were grown either in YPD or in YPD with 2 μg/mL doxycycline. The New England Biolabs Luna Universal One-Step RT-qPCR kit was used to measure gene expression levels. *ACT1* gene expression was used for the first normalization, yielding Δ*Ct* values. The Δ*Ct* values for *tet-POL2* and *tet-POL3* cultures grown in YPD were compared with wild-type cultures grown in YPD and for *tet-POL2* and *tet-POL3* cultures grown in YPD with doxycycline were compared with wild-type cultures grown in YPD and doxycycline to yield ΔΔ*Ct* values. Fold-change in gene expression was then calculated as 2^−ΔΔ^*^Ct^*. Gene expression levels of wild-type cultures were denoted as fold-change of 1, and *tet-POL2* and *tet-POL3* RNA levels were compared against them. Primers used for the qRT-PCR were as follows: ACT1-qPCR-Fwd: 5′ GGCTTCTTTGACTACCTTCCA 3′, ACT1-qPCR-Rev: 5′ AGAAACACTTGTGGTGAACGA 3′, POL2-qPCR-Fwd: 5′ CTACCGGAATCTGTCTTTCTGG 3′, POL2-qPCR-Rev: 5′ CGCTCTTTGATGAGGAGTGATAG 3′, POL3-qPCR-Fwd: 5′ GAGGGTGATTGGTCTCATACAG 3′, POL3-qPCR-Rev: 5′ TTGGATGACGGGATCGTATTC 3′.

Multiple *t*-tests were performed. *POL2* and *POL3* gene expressions in the *tet-POL2* and *tet-POL3* strains were compared with wild-type strains grown either with or without doxycycline. A false discovery rate correction was applied to obtain *q*-values.

### Whole genome and mutation analysis

Individual canavanine resistance (Can^R^) isolates were obtained for wild-type and *Drad1* isolates after treatment with either water or with 1% acetaldehyde. The colonies were streaked out to obtain pure cultures, and genomic DNA was extracted using the Zymo YeastStar genomic DNA isolation kit (Genesee Scientific). A total of 10 ng/μL DNA was used for library preparation with the Watchmaker DNA library prep with fragmentation (Watchmaker Genomics). Each sample was provided a unique dual index adapter (stubby adapter IDT, xGen UDI 10nt Primer Plate 1-4 IDT). Samples were pooled and sequenced on 1 lane of Illumina NovaSeq 6000 S4 PE 2 × 150 sequencing system. The resulting FASTQ files were aligned to the reference genome ySR128 (Roberts *et al.* 2012) using BWA mem (Li and Durbin 2009); duplicate reads were removed using Picard tools (http://broadinstitute.github.io/picard/), and mutations were called using VarScan2 using a variant allele frequency filter of 90% (Koboldt *et al.* 2009, 2012, 2013). The untreated original cultures were also sequenced. Mutations present in the untreated cultures and mutations common to 2 or more samples were marked as preexisting and removed.

TRInucleotide Mutation Signatures (TriMS) (Vijayraghavan *et al.* 2022) was used to further measure the enrichment of the gCn→A mutation signature in the acetaldehyde and water-treated isolates. Specifically, TriMS compares the total number of gCn→A mutations in a sample with the number of C→A changes in the same sample, as well as the number of cytosines and gcn motifs within 20 bp of a mutated residue. An enrichment greater than 1 is considered to be positively enriched. Our results are further tested statistically using a 1-sided Fisher’s exact test with the hypothesis that in samples with a positive enrichment, gCn→A mutations vs C→A mutations will have a higher value than gcn motifs vs cytosines in the background. The code for TRIMS is deposited in GitHub and can be accessed at https://github.com/SainiLabMUSC/TriMS.

## Results

### Acetaldehyde is not mutagenic in DNA repair proficient yeast strains

**Fig. 1.**
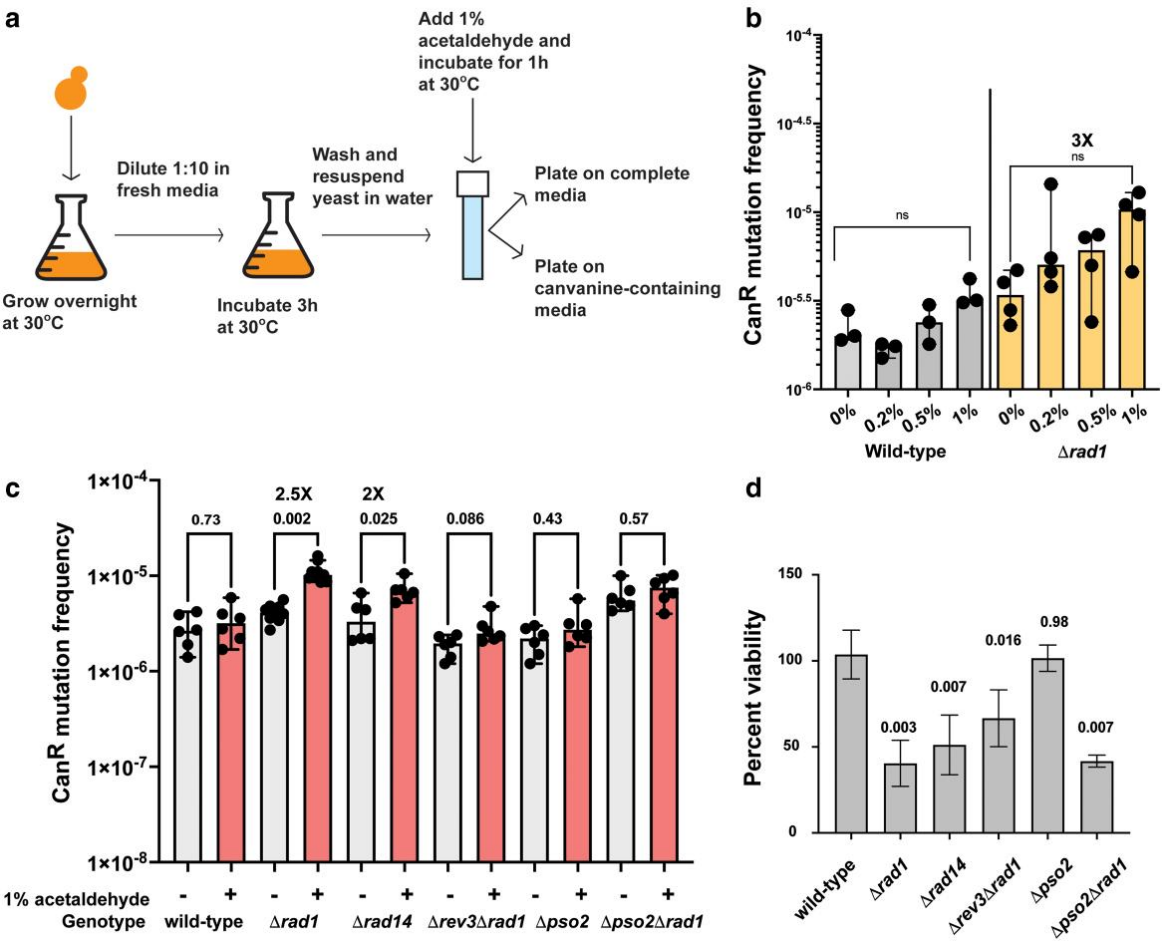
NER prevents acetaldehyde-induced mutagenesis in yeast. a) Schematics of the experiment (see *Materials and methods* for details). b) Pilot assay depicting mutation frequencies of wild-type and Drad1 strains treated with different concentrations of acetaldehyde. Bars indicate median mutation frequency, and error bars denote range. A Student’s t-test was performed to obtain P-values, and multiple testing was corrected using the Benjamini–Hochberg method. c) Mutation frequencies of yeast strains treated with no mutagen (−) or 1% acetaldehyde (+). Bars indicate median mutation frequencies, and error bars indicate 95% confidence intervals. Median and 95% confidence intervals are depicted. P-values were calculated using a Mann–Whitney U test. Benjamini–Hochberg corrected was applied to calculate the q-values accounting for multiple testing (see Supplementary Table 1). *q*-values and fold differences are indicated. d) Quantitative survival assay of yeast strains treated with acetaldehyde when compared with no mutagen. *P*-values were calculated using a Mann–Whitney U test. Multiple testing was corrected for by using the Benjamini–Hochberg method (see Supplementary Table 2). *q*-values are shown above the bars.

We previously demonstrated that acetaldehyde was highly mutagenic to single-stranded DNA in yeast (Vijayraghavan *et al.* 2022). However, various studies in bacteria, yeast, and iPSCs showed that acetaldehyde exposure was not mutagenic to cells (Dellarco 1988; Kucab *et al.* 2019; Voordeckers *et al.* 2020). To address this discrepancy, we cultured haploid yeast cultures overnight and subcultured into fresh media for 3 h to obtain actively dividing yeast cells (Fig. 1a). We hypothesized that yeast cultures with active DNA replication and transcription should consistently have single-stranded DNA available for acetaldehyde-induced mutagenesis. We incubated yeast cultures with varying concentrations of acetaldehyde and asked whether there was an impact on mutagenesis using plating assays (Fig. 1a). We did not observe any increase in Can^R^ mutation frequencies in the wild-type isolates treated with the different concentrations of acetaldehyde. Also, no changes in cellular viability were observed at 1% acetaldehyde when compared with the water-treated samples. These data corroborate the findings from others and demonstrate that acetaldehyde is likely not mutagenic to yeast with proficient DNA repair pathways (Fig. 1b–d; Supplementary Tables 1 and 2).

### Defects in NER increases acetaldehyde-induced mutation frequencies

Rad1 (ERCC4 in humans) is an endonuclease that is central to NER [reviewed in Prakash and Prakash (2000)]. We performed a pilot assay with 3–4 wild-type or *Drad1* isolates treated with varying concentrations of acetaldehyde as described above (Fig. 1). We saw a 3-fold increase in mutagenesis in the *Drad1* cultures treated with 1% acetaldehyde for 1 h in this pilot assay (Fig. 1b). We re-tested these cultures along with wild-type with a higher number of isolates and noted that *Drad1* strains had elevated acetaldehyde-induced mutation frequencies when compared with the untreated cultures (2.5-fold increase) (Fig. 1c). Additionally, we noted a decrease in cellular viability in these isolates upon acetaldehyde exposure (median viability 44%), indicating that NER is a major pathway for repair of acetaldehyde-induced DNA damage (Fig. 1c and d; Supplementary Tables 1 and 2).

To further validate our findings on the importance of NER in repairing acetaldehyde-induced DNA damage, we deleted the *RAD14* gene and analyzed mutation frequencies upon acetaldehyde exposure. Rad14 (XPA in humans) recognizes and binds DNA damage to promote NER (Prakash and Prakash 2000). *Drad14* strains treated with acetaldehyde demonstrated a similar increase in mutagenesis when compared with the untreated cultures (2-fold increase) (Fig. 1c; Supplementary Table 1). Acetaldehyde treatment led to a reduction in the viability of *Drad14* cultures to 46% when compared with the untreated cultures (Fig. 1c and d; Supplementary Table 2).

Previously, we also demonstrated that acetaldehyde adducts in single-stranded DNA are bypassed by TLS polymerases, leading to mutagenesis (Vijayraghavan *et al.* 2022). Rev3 is the catalytic subunit of DNA Polz and is essential for TLS in yeast [reviewed in Martin and Wood (2019)]. To determine if the increase in mutations seen in the *Drad1* strains upon treatment with acetaldehyde was dependent on TLS, we deleted *REV3* in the *Drad1* strain. Upon treatment, acetaldehyde mutation frequency was reduced to roughly wild-type levels in the *Δrev3Δrad1* strains, indicating that acetaldehyde-induced mutagenesis in the *Drad1* strains was dependent on Rev3/Polz (Fig. 1c; Supplementary Table 1). Our data demonstrate that unrepaired acetaldehyde-induced DNA adducts in the NER-deficient *Drad1* isolates are bypassed by TLS resulting in mutagenesis.

### DNA ICLs likely contribute to acetaldehyde mutagenesis

Among the various adducts induced by acetaldehyde, inter- and intrastrand crosslinks have been shown to be highly mutagenic (Matsuda *et al.* 1998; Brooks and Zakhari 2014; Balbo and Brooks 2015). In yeast, Pso2 is involved in mutagenic repair of DNA ICLs induced upon psoralen and ultraviolet radiation exposure. Consequently, *pso2* mutants demonstrate reduced mutation frequencies upon induction of DNA crosslinks (Cassier *et al.* 1980). Upon treatment, we did not observe any change in Can^R^ mutation frequencies in *Dpso2* yeast strains compared with the untreated controls. However, the increase in mutagenesis seen upon acetaldehyde treatment in the *Drad1* strain was abolished in the *Drad1Dpso2* double mutant strains (Fig. 1c; Supplementary Table 1). These data indicate that ICLs induced by acetaldehyde are repaired by NER and require Pso2 activity for TLS and mutagenesis.

### Acetaldehyde-induced gCn→A mutations are elevated in *Drad1* strains

**Fig. 2.**
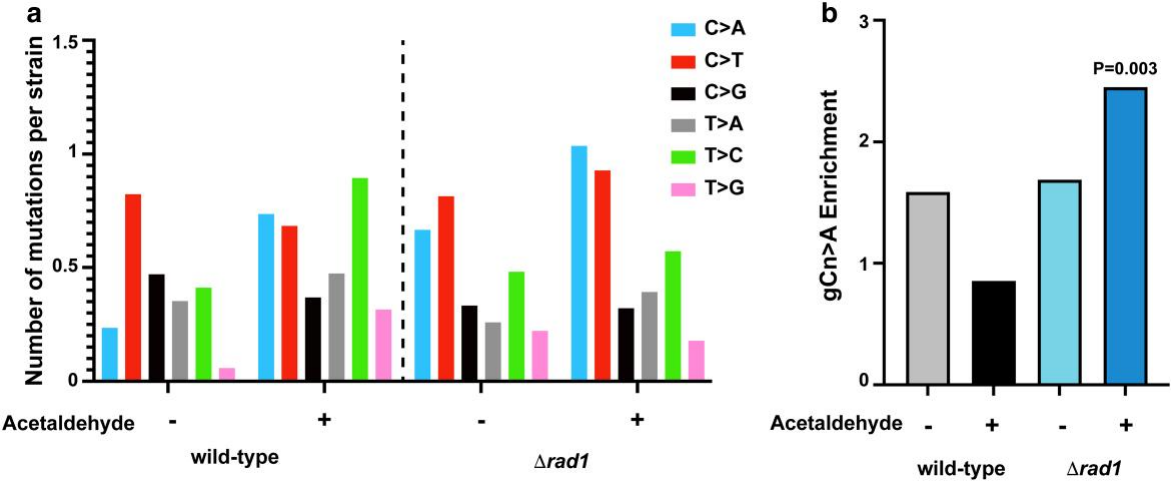
Acetaldehyde-induced mutation spectrum and signature in wild-type and *Drad1* isolates. a) The number of single base substitution (given substitution along with substitution of the complementary base) per isolate with acetaldehyde or no mutagen control are shown. Mutation spectra are plotted as pyrimidine changes, taking into consideration reverse complement for each base change. b) Enrichment of the acetaldehyde-specific gCn→A mutation signature in the yeast strains. *P*-value was calculated in TriMS using a Fisher’s exact test as described in the *Materials and methods.*

We further obtained genomic DNA from Can^R^ yeast isolates either after treatment with acetaldehyde or after treatment with water (untreated) and sequenced their genomes. We analyzed 17 Can^R^ wild-type yeast strains treated with water, 19 Can^R^ wild-type yeast strains treated with acetaldehyde, 27 Can^R^*Drad1* isolates treated with water, and 28 Can^R^*Drad1* isolates treated with acetaldehyde. Analysis of the mutation spectrum in these isolates demonstrated an increase in C→A changes upon acetaldehyde treatment compared with untreated strains, both in wild-type and *Drad1* strains (Fig. 2a and b; Supplementary Table 3). We then asked if the acetaldehyde-induced gCn→A mutation signature was elevated in the samples. We used the Trinucleotide Mutation Signature pipeline (TriMS) (Vijayraghavan *et al.* 2022) to determine if gCn→A changes were enriched in the strains. TriMS compares the number of gCn→A changes with the number of C→A changes in the same datasets, as well as the number of cytosines and the number of gcn motifs present in the background of each mutated residue. We note that acetaldehyde is a weak mutagen, and even in the *Drad1* isolates, we see a very small number of mutations induced by acetaldehyde. As such, we analyzed all mutations induced by acetaldehyde in each genotype as a single cohort of mutations. The low number of mutations does not allow us to detect signatures in individual isolates. We saw a statistically significant enrichment of the gCn→A signature only in the *Drad1* Can^R^ isolates obtained after treatment with acetaldehyde (Fig. 2b; Supplementary Table 3). The enrichment of the gCn→A signature strongly indicates that the increased mutation frequencies seen in the *Drad1* isolates were due to an increase in acetaldehyde-specific mutagenesis.

### DPC repair defects increase acetaldehyde mutation frequencies

**Fig. 3.**
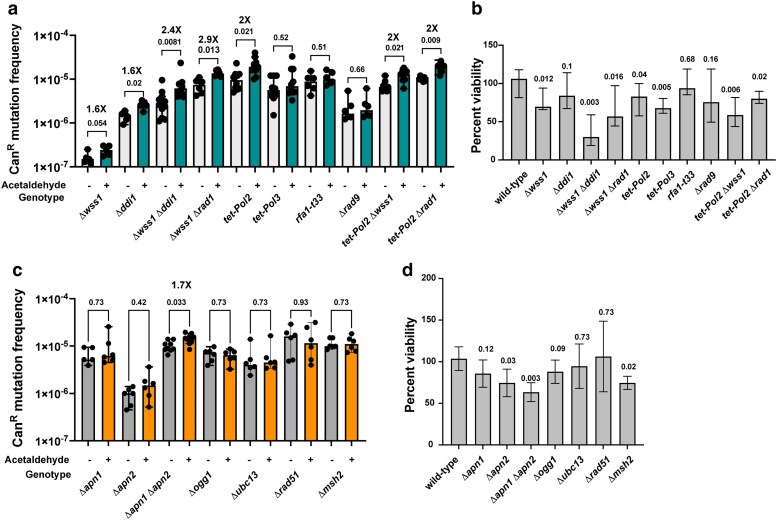
Multiple DNA repair pathways function to prevent acetaldehyde-induced mutagenesis. a) Mutation frequencies of yeast strains with defects in DPC repair or replication deficiencies. “+” indicates strains treated with 1% acetaldehyde, and “−” indicates strains treated with no mutagen. Median mutation frequencies are plotted, and error bars denote 95% confidence intervals. b) Quantitative survival assay of yeast strains with defects in DPC repair or replication deficiencies treated with acetaldehyde. Median and 95% confidence intervals are depicted. c) Mutation frequencies of BER-defective yeast strains, yeast strains defective in postreplicative repair (*Dubc13*), HR (*Drad51*), or MMR (*Dmsh2*). Median mutation frequencies are plotted, and error bars denote 95% confidence intervals. d) Viability of strains defective in BER, postreplicative repair (*Dubc13*), HR (*Drad51*), or MMR (*Dmsh2*). *P*-values were calculated using Mann–Whitney U test. Benjamini–Hochberg method was used for correction of multiple testing, and *q*-values are reported (see Supplementary Tables 1 and 3).

Wss1 (SPRTN in humans) and Ddi1 proteins are metalloproteases that have been shown to function in the removal of formaldehyde-induced DPCs (Stingele *et al.* 2014, 2016; Vaz *et al.* 2016; Reinking *et al.* 2020). Since acetaldehyde has also been shown to generate DPCS, we deleted the genes *WSS1* and/or *DDI1* in yeast. We found that compared with untreated controls, acetaldehyde-induced mutation frequencies were increased 1.6-fold in the *Dwss1* and 1.6-fold in *Dddi1* isolates, each (Fig. 3a). In the *Dwss1Dddi1* double mutant, we saw an additive increase in acetaldehyde-induced mutation frequency (2.4-fold) (Fig. 3a; Supplementary Table 1). We further noted that in contrast to the single deletion strains, the *Dwss1Dddi1* double mutant was highly sensitive to acetaldehyde, with a considerable loss of viability of the treated cultures (30%) compared with the untreated cells (Fig. 3b; Supplementary Table 2). Overall, we demonstrate that DPC repair is a major pathway for prevention of acetaldehyde-induced mutagenesis and cell death.

To further understand the interplay between DPC repair and NER, we generated the *Dwss1Drad1* isolate. Treatment of the double mutant with 1% acetaldehyde led to a 2.9-fold increase in mutagenesis compared with untreated strains (Fig. 3a). No further increase in mutation frequency was noted compared with the *Drad1* isolate treated with acetaldehyde. Our work points toward redundancy in the activities of DPC repair and NER in preventing acetaldehyde mutagenesis.

### Roles of DNA replication and checkpoint pathways in modulating acetaldehyde-induced mutagenesis

In *S. pombe*, it was shown that acetaldehyde leads to replication stress, which in turn activates the ataxia telangiectasia and Rad3-related (ATR)-dependent checkpoint (Noguchi *et al.* 2017). However, the impact of DNA replication defects on acetaldehyde-induced DNA damage and mutagenesis is not known. We downregulated the expression of the genes *POL2* (leading strand polymerase) and *POL3* (lagging strand polymerase). The promoters of both genes were replaced by the tetracycline-downregulatable promoter (*tetO7*) (Belli *et al.* 1998). In cultures grown in 2-μM doxycycline-containing media, we noted that expressions of POL2 and POL3 RNAs were markedly lower (Supplementary Fig. 1 and Table 4). Treatment of *tet-POL2* strains with doxycycline and acetaldehyde showed a 2-fold increase in mutagenesis in *CAN1.* On the other hand, acetaldehyde did not elevate mutation frequency in the *tet-POL3* strain (Fig. 3a; Supplementary Table 1). We noted that both *tet-POL2* and *tet-POL3* strains demonstrated mild increases in cell death upon treatment with acetaldehyde (Fig. 3b; Supplementary Table 2). Our data echo work in *Xenopus* egg extracts, which demonstrated that DPCs on the leading strand lead to replication stalling, and bypass of such lesions is dependent on the translesion polymerase Pol ζ (Duxin *et al.* 2014). Based on these data, it is possible that in strains with lower levels of Pol2, leading strand replication is compromised. When such defective replication machinery encounters an acetaldehyde-induced lesion on the leading strand, it is unable to bypass the damage error free, likely leading to elevated fork stalling and mutagenesis.

To determine if DPC repair or NER defects further elevate mutations in the *tet-POL2* strain, we generated double *tet-POL2 Dwss1* and *tet-POL2 Drad1* strains. Surprisingly, we did not see any further increase in mutagenesis in the double mutants compared with *tet-POL2* strains treated with acetaldehyde (Fig. 3a; Supplementary Table 1). Such epistasis is likely due to the additional role of Pol2 in repair-associated synthesis.

Our previous work showed that elevated ssDNA is targeted by acetaldehyde for mutagenesis. As such, we also assayed a hypomorphic mutation in the *RFA1* gene (t33; S373P). *RFA1* encodes a subunit of the heterotrimeric replication protein a that binds to ssDNA during replication. The *rfa1-t33* mutant carries a point mutation in the DNA binding domain, decreasing its association with ssDNA (Deng *et al.* 2014). Yeast strains carrying the *rfa1-t33* mutation were shown to have increased ssDNA-specific mutagenesis by *APOBEC3A* and *APOBEC3B* enzymes as well (Hoopes *et al.* 2016). Surprisingly, we did not see any increase in acetaldehyde-induced mutations in *rfa1-t33* strains (Fig. 3a; Supplementary Table 1). We hypothesize that either the transient nature of the ssDNA in *rfa1-t33* strains does not allow for sufficient acetaldehyde targeting or that efficient repair abrogates such mutagenesis.

Finally, in *S. pombe*, acetaldehyde was shown to elicit ATR-dependent checkpoint activation and cell cycle arrest (Noguchi *et al.* 2017). As such, we deleted the *RAD9* gene in yeast responsible for DNA damage-induced checkpoint activation and cell cycle arrest. We did not see any increase in acetaldehyde-induced mutagenesis in these strains, indicating that checkpoint activation was not essential for mutagenesis (Fig. 3a; Supplementary Table 1).

### Other DNA repair pathways involved in preventing acetaldehyde-induced mutagenesis

Acetaldehyde exposure has also been linked to increased reactive oxygen species production [reviewed in Setshedi *et al.* (2010) and Voulgaridou *et al.* (2011)]. Such oxidative damage can lead to the accumulation of 8-oxo-guanine moieties leading to G→T/C→A mutations. 8-oxo-guanine residues are removed by the Ogg1 glycosylase, and the resulting abasic sites are repaired via the activity of the BER enzymes Apn1 and Apn2 (Ramotar *et al.* 1991; Sandigursky *et al.* 1997; Shinmura and Yokota 2001; Boiteux and Guillet 2004). Deletion of *OGG1* and treatment with 1% acetaldehyde did not result in any increase in mutagenesis in yeast (Fig. 3c; Supplementary Table 1). On the other hand, deletion of *APN1* or *APN2*, individually, showed a slight increase in Can^R^ mutagenesis. This increase was not statistically significant. However, we noted that the double *Dapn1Dapn2* mutants had higher mutation frequencies upon treatment with acetaldehyde (1.6-fold increase) (Fig. 3c; Supplementary Table 1). In addition, we saw a decrease in cellular viability of the *Dapn1Dapn2* mutants to 67% upon treatment with acetaldehyde as compared with the wild-type isolates (Fig. 3d; Supplementary Table 2). As such, we conclude that while acetaldehyde does not appear to contribute to oxidative damage and accumulation of 8-oxo-guanine residues in our system, BER is likely involved in the repair of a minor subset of acetaldehyde lesions.

To test other DNA repair pathways that may be involved in the repair of acetaldehyde-induced DNA damage in yeast, we deleted genes involved in postreplication repair (PRR) (*UBC13*) (Brusky *et al.* 2000), MMR (*MSH2*) (Harfe and Jinks-Robertson 2000), and HR (*RAD51*) (Symington 2002), respectively. We did not see any increase in acetaldehyde-induced mutation frequencies in these strains (Fig. 3c; Supplementary Table 1). We also did not detect any loss in viability of these mutants upon treatment with acetaldehyde (Fig. 3d; Supplementary Table 1), indicating that these pathways likely do not function as primary mechanisms to avoid acetaldehyde-induced DNA damage.

## Discussion

**Fig. 4.**
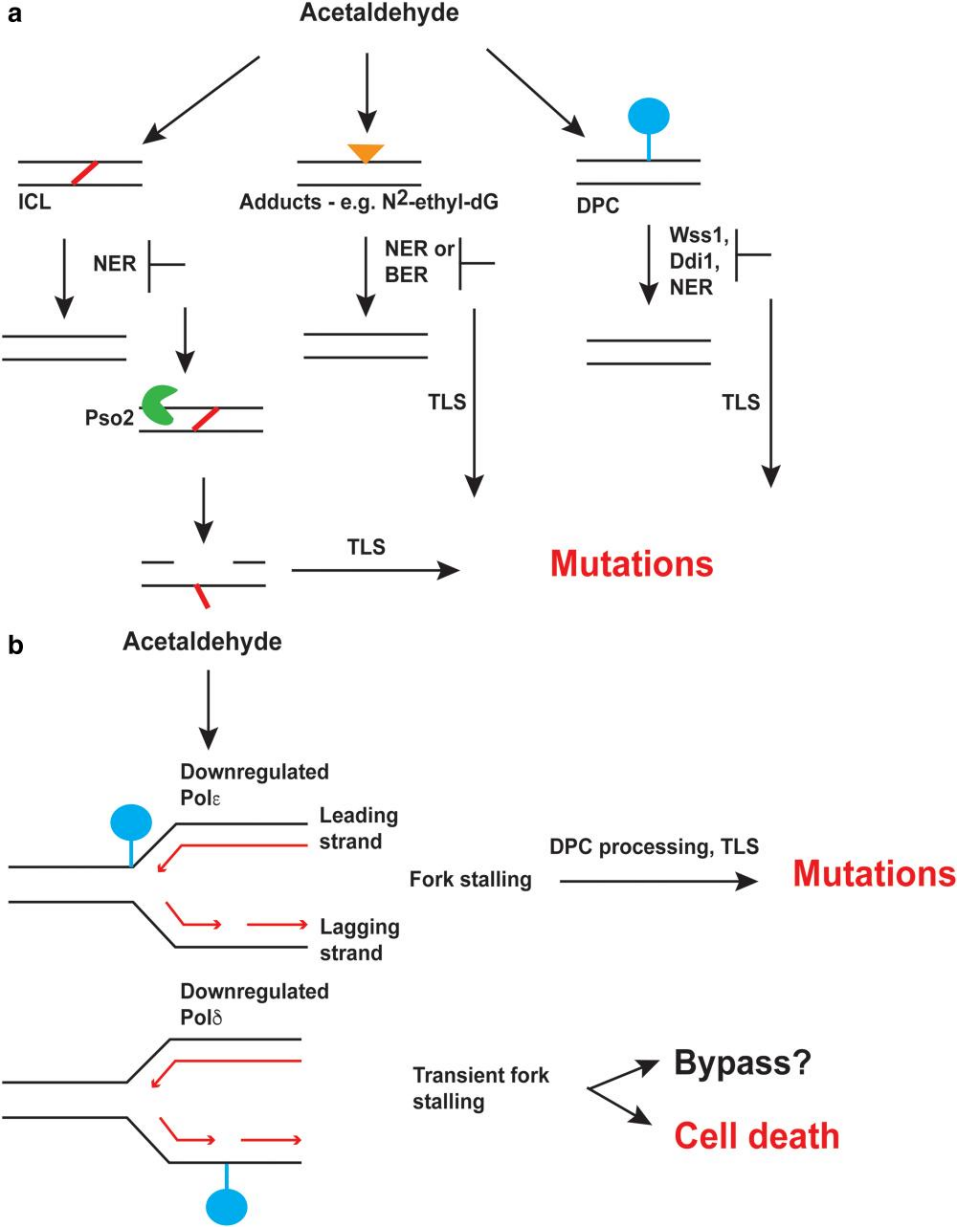
Model of a) DNA repair and b) defective DNA replication pathways that alter acetaldehyde-induced mutagenesis. Acetaldehyde-induced ICLs are shown as a slanted line between two DNA strands, DNA adducts are shown as a triangle, and DPCs are shown as a circle connected to DNA. The different DNA repair enzymes and pathways that likely function to prevent or promote mutagenesis by acting on these adducts are shown.

In this study, we demonstrate that acetaldehyde-induced DNA damage is repaired by multiple pathways to prevent mutagenesis. Defects in NER, DPC repair, BER, and ICL repair were found to alter acetaldehyde-induced mutation frequencies in yeast (Fig. 4). Interestingly, similar to previous studies that showed that acetaldehyde was not mutagenic in DNA repair proficient human iPSCs, *Salmonella* strains, and yeast strains (Dellarco 1988; Kucab *et al.* 2019; Voordeckers *et al.* 2020), we did not observe elevated acetaldehyde-induced mutagenesis in wild-type yeast strains. This indicates that DNA repair pathways are highly efficient at removing acetaldehyde lesions in the genome.

The deployment of multiple DNA repair pathways to prevent acetaldehyde-induced mutagenesis signifies the induction of different types of DNA lesions generated upon acetaldehyde exposure in cells (Fig. 4). Acetaldehyde is known to form 1,*N*^2^-propano-2′-deoxyguanosine (Matsuda *et al.* 1998; Brooks and Theruvathu 2005; Setshedi *et al.* 2010; Brooks and Zakhari 2014; Balbo and Brooks 2015; Sonohara *et al.* 2019), and these lesions have been shown to block DNA replication (Noguchi *et al.* 2017; Tsuruta *et al.* 2020). Such guanine lesions are likely repaired via NER. As such, deletion of *RAD1* or *RAD14* prevents excision of these lesions, causing replication defects, recruitment of TLS, and mutagenic bypass of the lesion.

Our data further elaborate the molecular mechanism underlying acetaldehyde-induced intra- and inter-strand crosslinks. Prior studies have shown that acetaldehyde induces G-G ICLs (Matsuda *et al.* 1998; Sonohara *et al.* 2019). The signature motif associated with acetaldehyde gCn/nGc has 2 guanine residues on opposite strands near each other, which are potentially conducive to ICL formation. Because NER is implicated in ICL repair [reviewed in Deans and West (2011)], *Δrad1* strains with defective NER likely have persistent ICLs that cause replication fork blocking. *Saccharomyces cerevisiae* predominantly uses Pso2 along with the helicase Hrq1 to endonucleolytically cleave DNA and unhook the ICL allowing for recruitment of a TLS polymerase and gap filling across the lesion (Rogers *et al.* 2020). In concordance with this model, we demonstrated that in *Drad1Dpso2* strains, acetaldehyde-induced mutagenesis is abrogated, likely due to the lack of TLS recruitment to the lesion.

Acetaldehyde exposure can also lead to the formation of DPCs, which can further block DNA replication and lead to double-strand breaks and genome instability. Studies using formaldehyde have demonstrated that DPCs are primarily repaired using the metalloproteases Wss1 (functional homolog of SPRTN) and Ddi1 in yeast (Stingele *et al.* 2014). Similarly, work on *S. pombe* strains demonstrated that the Wss1 metalloprotease was responsible for DPC resolution and deletion of *WSS1* sensitizes cells to acetaldehyde exposure (Noguchi *et al.* 2017). Here, our work demonstrates that both Wss1 and Ddi1 act redundantly to repair DPCs and prevent acetaldehyde-induced mutagenesis in yeast. Overall, our work and the studies in *S. pombe* together demonstrate that acetaldehyde-induced DPCs are a major source of genome instability. SPRTN deficiencies in humans and in mice have been linked to elevated liver cancers (Lessel *et al.* 2014; Batel *et al.* 2024). Since acetaldehyde-induced DNA damage has been implicated in liver cancers (Wang *et al.* 2020; Vijayraghavan *et al.* 2022), it is reasonable to assume that DPCs from endogenous acetaldehyde contribute to mutagenesis and genome instability in liver cancers.

NER has been implicated in the removal of DPCs in bacterial studies (Nishioka 1973; Takahashi *et al.* 1985), yeast (de Graaf *et al.* 2009), and mammalian cells (Baker *et al.* 2007). However, due to size constraints, NER is unable to remove very large DPCs and therefore relies on the proteolytic activity of Wss1 for their removal. In the absence of Wss1, either Ddi1 or the proteasomal complex likely functions to degrade the protein, allowing NER to fully repair the lesion (Nakano *et al.* 2009). Further, in *Caenorhabditis elegans*, loss of NER does not increase the sensitivity of SPRTN-defective worms to DPC induction by formaldehyde (Stingele *et al.* 2016). In keeping with these observations, we show that *Drad1* strains are epistatic to *Drad1 Dwss1* in modulating acetaldehyde mutagenesis, demonstrating that NER is likely involved in the repair of multiple lesions induced by acetaldehyde, including DPCs.

DPC repair is coupled to DNA replication. Using *Xenopus* egg extracts, it was shown that DPCs on the leading strand cause the replication fork to stall. The replicative DNA helicase CMG (CDC45, MCM2-7, GINS) can bypass the DPC, the replicative polymerase approaches the DPC, and Wss1/SPRTN is then recruited to the DPC (Duxin *et al.* 2014; Sparks *et al.* 2019). Bypass of the remaining peptide crosslink requires DNA polymerase ζ (Duxin *et al.* 2014; Sparks *et al.* 2019). Interestingly, DPCs on the lagging strand do not lead to similar fork stalling (Duxin *et al.* 2014). Finally, leading strand extension past an intact DPC is much slower than lagging strand extension past a DPC (Sparks *et al.* 2019). In sum, these data demonstrate an intricate relationship between DPCs and the replication machinery, with mechanistic differences between leading and lagging strand bypass of DPCs. In agreement with prior data, we demonstrate that downregulation of the leading strand polymerase, but not the lagging strand polymerase, leads to increased acetaldehyde-induced mutagenesis. Moreover, the epistasis of the *tet-Pol2* isolate with *Dwss1* mutations indicates that Wss1-initiated DPC repair is nonfunctional in these strains. We posit that downregulation of the leading strand polymerase likely impacts recruitment of Wss1 to the DPC, preventing its removal, thereby culminating in increased acetaldehyde mutagenesis. However, how DPCs on the lagging strand are repaired or bypassed in strains with Pol3 deficiencies is still unclear.

Previous reports have implicated ethanol consumption and acetaldehyde in generating oxidative stress. As such, we would anticipate that acetaldehyde treatment would lead to an increase in 8-oxo-guanine levels, which is predominantly repaired via BER. Unrepaired 8-oxo-guanines in DNA are bypassed via TLS and culminate in G→A mutations. Removal of *OGG1* did not cause elevated acetaldehyde mutations. We conclude that the gCn→A mutations are not a product of erroneous bypass of unrepaired 8-oxo-guanines. BER has previously been implicated in tolerance of acetaldehyde in *S. pombe* (Noguchi *et al*. 2017). In combination with prior studies, our data suggest that BER likely functions to remove DNA base adducts formed by acetaldehyde.

Finally, HR has been previously shown to impact the sensitivity of *S. pombe* to acetaldehyde (Noguchi *et al.* 2017). Further, Chinese Hamster Ovary cells deficient in the HR factor *RAD51D* were sensitive to acetaldehyde and demonstrated increased chromosomal aberrations (Mechilli *et al.* 2008). However, we did not see increased acetaldehyde-induced mutation frequencies in *Drad51* isolates. It is possible that HR or PRR serve as backup pathways to mitigate acetaldehyde genotoxicity and mutagenesis when other primary pathways (NER, DPC repair, ICL repair) are absent. Further, our assay is built to detect point mutations that inactivate *CAN1*, but not other genotoxic effects such as double-strand breaks and gross chromosomal rearrangements. It remains possible that pathways like HR and PRR function to limit such macroscale genotoxic events. A further limitation to our study is that our assay relies on subjecting yeast cultures to acute acetaldehyde exposure (i.e. short treatment time, high concentrations) as opposed to chronic exposure (i.e. constant exposure in media, low concentrations), largely owing to the extremely volatile nature of the chemical (acetaldehyde boiling point = 68.36°F/20.2°C). As such, DNA repair pathways that are functional during replication may be underrepresented in our assays. Finally, we also note that most of the pathways that alter acetaldehyde mutagenesis also led to reduction in cellular viability upon acetaldehyde exposure. It is possible that such viability decrease also impacts our ability to detect mutagenesis. Further investigation is required to precisely determine the role of additional repair pathways and DNA replication–associated mechanisms in maintaining genome stability upon acetaldehyde exposure.

Acetaldehyde is a known carcinogen and a DNA-damaging agent. We previously showed that an acetaldehyde-specific mutation signature can be detected in cancers, indicating that it is a source of DNA damage and mutagenesis in tumors (Vijayraghavan *et al.* 2022). Our work demonstrates that defects in a variety of DNA repair pathways can lead to elevated mutagenesis upon acetaldehyde exposure in yeast and in liver cancers, thereby highlighting the various types of DNA damage induced by acetaldehyde exposure. Considering that acetaldehyde is both an endogenous and environmental carcinogen, understanding the molecular mechanisms that alter acetaldehyde mutation rates is essential to enable determination of “at-risk” individuals susceptible to acetaldehyde-induced cancers.

## Supplementary Material

iyae213_Supplementary_Data

## Data Availability

The yeast strains used in the study are available upon request. Raw FASTQ sequence files from whole-genome sequencing of yeast samples have been deposited to the Sequence Read Archives (SRA) database and are accessible under PRJNA1065299. Sequence for the reference yeast genome used in this study (ySR128) is accessible on SRA under PRJNA524644. Source code for TriMS is available on GitHub (https://github.com/SainiLabMUSC/TriMS). Supplemental material available at GENETICS online.
