# Supplementary material for: Multiple DNA repair pathways prevent acetaldehyde-induced mutagenesis in yeast
Source: Genetics. 2024 Dec 21;229(4):iyae213. doi: 10.1093/genetics/iyae213 (PMC12005267; doi:10.1093/genetics/iyae213)
Supplement: iyae213_Supplementary_Data [file iyae213_supplementary_data.zip › Supplemental_Legends_GENETICS-2024-307707.docx]

**Supplemental Legends**

**Supplemental Figures**

**Figure S1.** Gene expression levels of wild-type and tetracycline downregulated polymerases. Gene expression levels were monitored in media with 2μM doxycycline or without doxycycline. Expression was compared against the genes under their endogenous promoters in the same media. Mean expression levels and standard error of the mean are depicted. * = Q-value < 0.001 using a multiple unpaired t tests. Benjamini Hoechberg method was applied to correct for multiple testing.

**Supplemental Tables**

**Table S1.** Data table for Figures 1B, 1C, 3A, 3C showing *Can^R^* mutation frequencies in yeast treated with acetaldehyde.

**Table S2**. Source data for Figures 1D, 3B and 3D showing viability of strains treated with acetaldehyde as compared to untreated strains.

**Table S3.** Data for Figure 2. Whole genome sequencing data from yeast strains in this study. S3A - Mutation list from whole genome sequenced yeast strains; S3B - Mutation spectrum of acetaldehyde and water-treated isolates; S3C – gCn🡪A mutation signature analysis using TriMS.

**Table S4.** Source data for quantitative reverse transcriptase PCR to determine gene expression levels of *POL2* and *POL3.*

**Table S5.** Yeast strains used in this study.
