# Supplementary figures and images for: Multiple DNA repair pathways prevent acetaldehyde-induced mutagenesis in yeast
Source: Genetics. 2024 Dec 21;229(4):iyae213. doi: 10.1093/genetics/iyae213 (PMC12005267; doi:10.1093/genetics/iyae213)

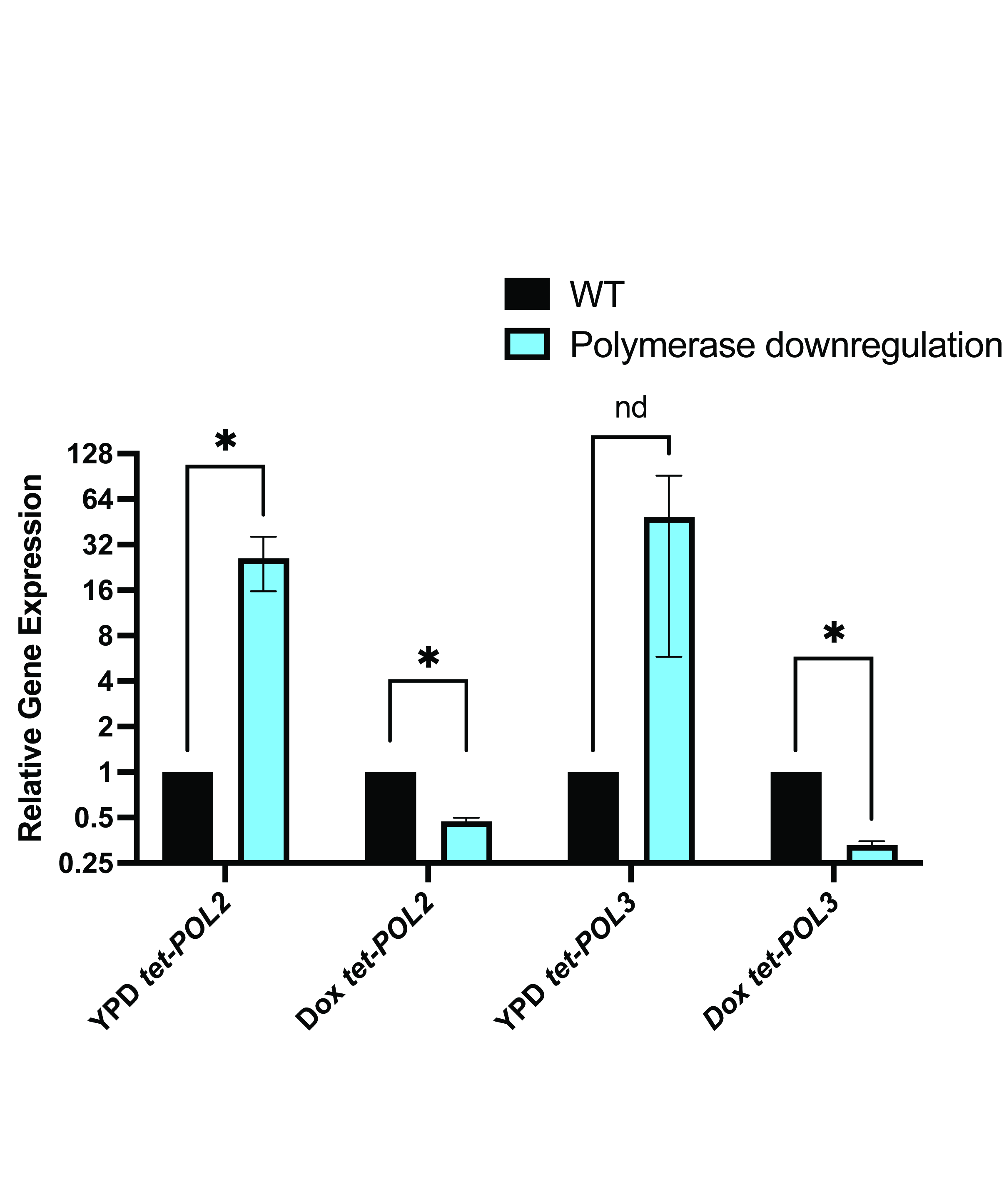

Supplement: iyae213_Supplementary_Data [file iyae213_supplementary_data.zip › Figure_S1_GENETICS-2024-307707.tif]
